# Purtscher‐Like Retinopathy in a Young Male With Renal Failure Following Bodybuilding Supplement Overuse: A Case Report

**DOI:** 10.1155/carm/7181385

**Published:** 2026-02-05

**Authors:** Sahel Khazaei, Hooman Khazaei

**Affiliations:** ^1^ Eye Research Center, Mashhad University of Medical Sciences, Mashhad, Khorasan Razavi, Iran, mums.ac.ir; ^2^ Department of Ophthalmology, Kurdistan University of Medical Sciences, Sanandaj, Kurdistan, Iran, muk.ac.ir

**Keywords:** acute kidney injury, anabolic androgenic steroids, dietary supplement, hypertensive crisis, kidney diseases, Purtscher-like retinopathy, renal insufficiency

## Abstract

Purtscher‐like retinopathy is a rare microvascular retinal disorder characterized by sudden visual loss and distinctive fundoscopic findings, often associated with systemic vascular insults. We report a 31‐year‐old male presenting with bilateral sudden blurred vision, hypertensive crisis, and renal failure following excessive intake of anabolic steroids, amino acids, and creatine supplements. Fundoscopy revealed Purtscher flecken, cotton‐wool spots, and intraretinal hemorrhages, consistent with Purtscher‐like retinopathy. Spectral‐domain OCT demonstrated inner retinal hyperreflectivity and fluid accumulation. The patient’s systemic condition was managed with antihypertensive therapy and hemodialysis; however, he left prematurely, limiting follow‐up. This case underscores the importance of recognizing ocular findings as early indicators of systemic vascular compromise in young patients with anabolic steroid and supplement abuse, highlighting the systemic risks of such overuse and the critical role of early diagnosis and interdisciplinary management.

## 1. Introduction

Purtscher‐like retinopathy is a rare microvascular retinal disorder characterized by occlusion of small retinal vessels, leading to distinctive fundoscopic findings such as cotton‐wool spots, intraretinal hemorrhages, and polygonal patches of retinal whitening known as Purtscher flecken. Unlike classic Purtscher’s retinopathy, which is typically associated with traumatic injuries involving the head or thorax, Purtscher‐like retinopathy occurs in the absence of trauma and is linked to various systemic conditions. These include acute pancreatitis, connective tissue diseases, renal failure, and other inflammatory or ischemic states [1]. The condition is thought to result from microembolization, complement activation, or endothelial damage leading to retinal ischemia, although the precise pathogenic mechanisms remain incompletely understood [2]. While its incidence is considered low, it may be underdiagnosed due to the often‐asymptomatic nature of early cases [1]. In recent years, emerging associations have highlighted the potential for Purtscher‐like retinopathy to occur secondary to systemic vascular insults, including those related to medication toxicity or systemic overuse [3, 4]. This report presents a case of Purtscher‐like retinopathy developing in a young male following renal failure induced by excessive use of bodybuilding supplements, underscoring the importance of recognizing this rare but significant ocular complication in systemic conditions related to supplement overuse.

## 2. Case Report

**FIGURE 2:**
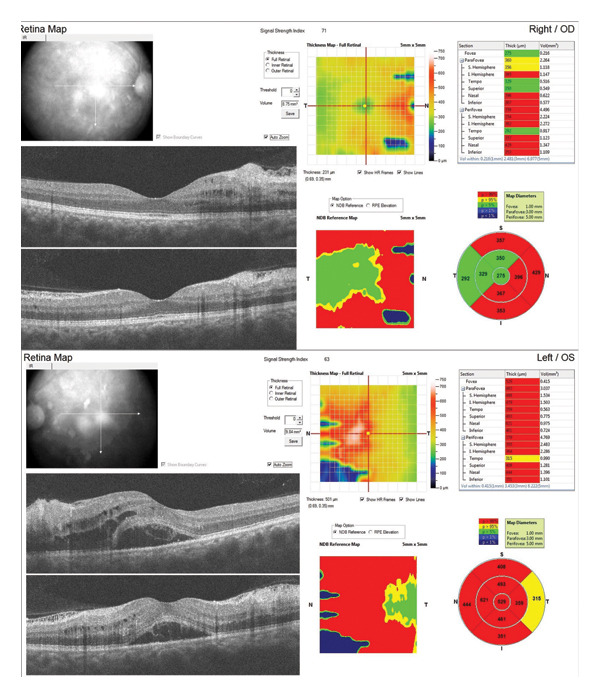
Infrared reflectance images of both eyes, and macular SD‐OCT of both eyes showing irregularity and wrinkling of the retinal surface, retinal thickening, subretinal and intraretinal fluid, and hyperreflectivity in the inner retinal layers corresponding to cotton‐wool spots.

A 31‐year‐old man was referred to our eye clinic due to sudden blurred vision in both eyes. He also complained of a headache. The patient reported a history of excessive consumption of bodybuilding supplements, including anabolic–androgenic steroids (nandrolone decanoate), L‐glutamine, and creatine, over the past 6 months. He denied any prior ocular or systemic illnesses. Visual acuity was 20/30 in the left eye and 20/25 in the right eye. Intraocular pressure measured 14 mm Hg in the right eye and 13 mm Hg in the left eye. Anterior segment examination of both eyes was unremarkable. Fundoscopy revealed subretinal fluid in the macular region, Purtscher flecken, extensive cotton‐wool spots all over the posterior pole involving the peripapillary area, and a small number of intraretinal hemorrhages surrounding a normal optic disc bilaterally, as well as vascular tortuosity (Figure 1). Spectral‐domain optical coherence tomography (SD‐OCT) findings included retinal surface irregularity with wrinkling, increased retinal thickness, and the accumulation of subretinal and intraretinal fluid. Hyperreflectivity of the inner retinal layers was evident in areas corresponding to cotton‐wool spots (Figure 2). During the systemic physical examination, high blood pressure was noted (210/150 mmHg). Laboratory investigations indicated renal failure with a glomerular filtration rate of 6.9 mL/min/1.73 m^2^ using the CKD‐EPI equation (Table 1). Autoimmune workup, including antinuclear antibody (ANA), anti–double‐stranded DNA (anti‐dsDNA), complement levels (C3 and C4), and antiphospholipid antibodies, was negative, reducing suspicion for connective tissue diseases such as systemic lupus erythematosus (SLE). Amylase and lipase levels were normal, and fasting glucose and HbA1c were within normal limits. Urine toxicology was negative for amphetamine, methamphetamine, marijuana, methadone, and morphine. Lactate dehydrogenase (LDH) was mildly elevated at 808 U/L and creatine phosphokinase (CPK) at 2390 U/L, consistent with possible rhabdomyolysis from supplement overuse but normalizing posthydration. The patient was admitted to the intensive care unit for aggressive antihypertensive therapy with intravenous labetalol (initial bolus 20 mg, followed by infusion titrated to maintain BP < 160/100 mmHg) and oral amlodipine (10 mg daily), alongside the initiation of hemodialysis (4‐h sessions via temporary catheter, with a Kt/V target of 1.2–1.4 per session) for acute kidney injury (AKI) with uremic symptoms, metabolic acidosis (pH 7.25, bicarbonate 15 mEq/L), and oliguria (< 0.5 mL/kg/hr), meeting the KDIGO Stage 3 criteria. His supplement use was stopped. No local ocular treatments were administered. Abdominopelvic ultrasonography showed normal size kidneys and normal thickness of parenchyma but increased parenchymal echogenicity. A tardus parvus waveform pattern was seen in the left intraparenchymal arteries, which was in favor of intraparenchymal arterial stenosis. Blood pressure was gradually stabilized. Over the following days, the patient reported subjective improvement in vision. However, the patient left the hospital prematurely, before completing the full treatment plan. Follow‐up was attempted via telephone. At 1 week postdischarge, the patient reported continued subjective visual improvement and stable blood pressure on oral medication. At 3 months, best‐corrected visual acuity was measured by an optometrist in his city of residence as 20/20 bilaterally. However, due to the patient’s early discharge and limited contact, renal function and blood pressure status were not documented at follow‐up. No serial fundus photographs or OCT scans were obtained due to loss to follow‐up.

FIGURE 1Color fundus photography of both eyes (a, b), showing subretinal fluid in the macular region, Purtscher flecken, cotton‐wool spots all over the posterior pole involving the peripapillary area, and a small number of intraretinal hemorrhages surrounding a normal optic disc bilaterally, as well as vascular tortuosity.

**Figure:**
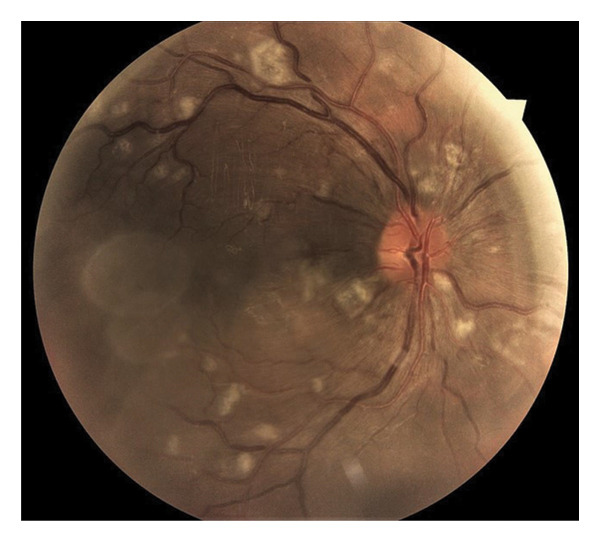
(a)

**Figure:**
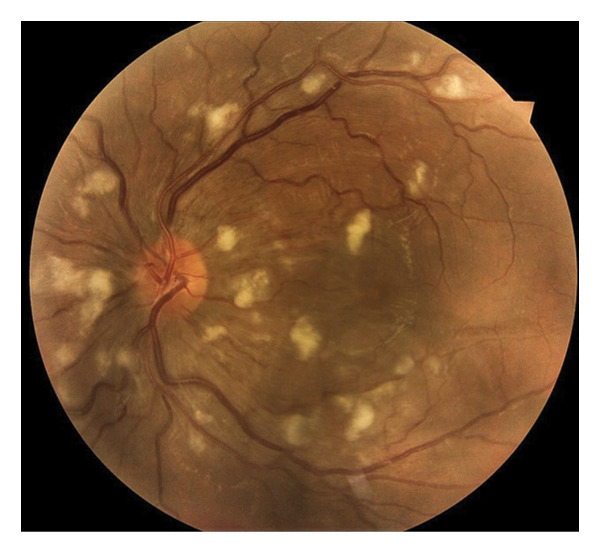
(b)

**TABLE 1:** Laboratory test results.

Test	Patient values on admission	Patient values, last day of admission	Normal values (ranges)
Hemoglobin (g/dL)	10.4		14–18
Hematocrit (%)	30.8		39–52
MCV (fL)	97		80–96
MCH (Pg)	33		26–34
Leukocytes (10^3^/mL)	7.4		4–10
Platelets (10^3^/mL)	135		150–450
BUN (mg/dL)	26	16	8.87–20.50
Creatinine (mg/dL)	9.0	6.3	0.7–1.3
Serum Ca (mg/dL)	7.4		8.6–10.3
Serum Mg (mg/dL)	2.5		1.8–2.6
PTH (Pg/mL)	114.4		15–65
Serum Na (meq/L)	144.3	145	135–145
Serum K (meq/L)	4.2	5	3.5–5
FBS (mg/dL)	99	93	70–140
SGOT (U/L)	33		< 37
SGPT (U/L)	46		< 41
Alk.P (U/L)	178		80–306
CPK (U/L)	2390		≤ 171
LDH (Iu/L)	808		235–470
Amylase (u/L)	78		< 90
Urinalysis (mg/dL)			
Blood	2+		Negative
RBC	7‐8/high power field		
Urine volume/24 h (mL)	1000		800–1800
Urine protein/24 h (mg)	2299		40–150
Urine creatinine/24 h (mg)	809		800–2000

*Note:* Ca, calcium; CPK, creatine phosphokinase; K, potassium; LDH, lactate dehydrogenase; Mg, magnesium; Na, sodium; PTH, parathyroid hormone.

Abbreviations: Alk.P, alkaline phosphatase; BUN, blood urea nitrogen; FBS, fasting blood sugar; MCH, mean corpuscular hemoglobin; MCV, mean corpuscular volume; RBC, red blood cell; SGOT, serum glutamic‐oxaloacetic transaminase; SGPT, serum glutamic pyruvic transaminase.

## 3. Discussion

Purtscher‐like retinopathy is a rare and intriguing microvascular retinal disorder characterized by distinctive fundoscopic findings such as Purtscher flecken, cotton‐wool spots, and intraretinal hemorrhages, typically in the absence of significant trauma [2]. While classic Purtscher’s retinopathy is closely linked to traumatic injuries involving the head or thorax, Purtscher‐like retinopathy extends its association to a variety of systemic illnesses and vascular insults, emphasizing its multifactorial and systemic nature [2, 5, 6].

The increasing adoption of muscle‐building and fat‐reducing practices among young people and athletes is often not supported by robust scientific knowledge. This situation has stimulated scholarly attention toward evaluating the prevalence of dietary supplements and androgenic–anabolic steroids and examining the range of their potential side effects [7].

In this case, a 31‐year‐old male presented with bilateral sudden blurred vision, with fundoscopic examination revealing hallmark features of Purtscher‐like retinopathy. The diagnostic criteria for Purtscher retinopathy, as outlined by Miguel et al. [1], involve the identification of at least three of the following features: Purtscher flecken, a low‐to‐moderate number of retinal hemorrhages, cotton‐wool spots (as presented in our patient), and a probable or plausible underlying etiology (renal failure in this case). The systemic findings of hypertension and renal failure, along with a history of excessive bodybuilding supplement intake, are noteworthy. Similar cases in the literature reinforce this association: Renal impairment from nephrotic syndrome or chronic kidney disease has been linked to Purtscher‐like retinopathy via complement‐mediated endothelial damage and microembolization [8–10]. Notably, associations with supplement overuse remain uncommon. No prior reports directly link anabolic steroids to Purtscher‐like retinopathy, highlighting the novelty of this presentation and alerting clinicians to potential iatrogenic risks.

While the presentation aligns with Purtscher‐like retinopathy secondary to supplement‐induced renal failure and hypertension, a broad differential is essential in nontraumatic cases. Systemic hypertension alone can cause retinopathy with cotton‐wool spots and hemorrhages, but the presence of Purtscher flecken suggests a more specific microangiopathy. SLE was considered due to potential complement‐mediated vascular damage and renal involvement, but negative autoimmune serologies (ANA, anti‐dsDNA, and C3/C4) made this unlikely. Acute pancreatitis, a known trigger, was excluded by normal amylase/lipase levels and the patient’s physical exam. Diabetes mellitus could contribute via microvascular ischemia, but normal glucose and HbA1c ruled it out. Other considerations include medication toxicity (e.g., from supplements) and thrombotic microangiopathies, but the history and labs supported an iatrogenic etiology.

The patient’s excessive consumption of bodybuilding supplements, such as anabolic–androgenic steroids and creatine, has been associated with various systemic adverse effects. Evidence suggests that excessive bodybuilding supplement use may play a role in the development of hypertension and renal dysfunction through several mechanisms, including AKI, chronic kidney disease, and glomerular toxicity [8]. These adverse outcomes are linked to stimulation of the renin–angiotensin–aldosterone system, augmented endothelin production, increased generation of reactive oxygen species, enhanced expression of profibrotic and proapoptotic mediators (e.g., TGF‐β1), and activation of inflammatory cytokines such as TNF‐α, IL‐1b, and IL‐6 [11].

According to the limited follow‐up period of our patient and the lack of baseline creatinine level, differentiating between acute renal injury and chronic kidney disease was difficult. Some findings such as normocytic normochromic anemia and the presence of hypocalcemia and hyperphosphatemia are in favor of a chronic process. However, the oliguria (< 0.5 mL/kg/h) is indicative of AKI according to the KDIGO clinical practice guideline for AKI [12]. Considering all aspects of the clinical manifestation of the present case, an acute‐on‐chronic kidney injury is assumed.

One of the other potential diagnoses that should be considered for this patient is medication‐induced interstitial nephritis. However, the absence of white blood cell casts, eosinophilia, and increased inflammatory markers in the blood make this diagnosis less likely, although it cannot be definitively ruled out without performing a kidney biopsy.

The use of anabolic–androgenic steroids in supraphysiological doses has been linked to abnormal plasma lipoprotein profiles, which can accelerate the development of atherosclerosis [13]. Furthermore, excessive levels of anabolic–androgenic steroids can activate androgenic receptors, cell membrane receptors, and downstream signaling pathways, thereby stimulating the renin–angiotensin–aldosterone system and contributing to the development of hypertension [11]. The abuse of anabolic–androgenic steroids has been repeatedly associated with an elevated risk of thrombosis, which is a presumed mechanism in the development of Purtscher‐like retinopathy [11]. However, the existing literature has not adequately elucidated the relationship between anabolic–androgenic steroids abuse and thrombotic events, with only limited reports addressing actual thrombotic outcomes [14]. Similar to other tissues and organs, oxidative stress, apoptosis, and inflammation play a crucial role in kidney injury. Prolonged exposure to nandrolone in animal models has been demonstrated to induce dose‐dependent oxidative stress and damage to the kidneys [11].

The elevated blood pressure and renal failure suggest a possible link between supplement overuse and systemic vascular compromise, which may have contributed to the retinal microvascular ischemia observed.

The pathogenesis of Purtscher‐like retinopathy remains incompletely understood but is believed to involve microembolization, complement activation, endothelial damage, and subsequent retinal ischemia [1]. In systemic conditions such as renal failure, hypertension, and inflammatory states, these mechanisms can be exacerbated, leading to retinal microvascular occlusion. The association with systemic vascular insults underscores the importance of recognizing this retinal manifestation as a potential indicator of underlying systemic pathology.

The case highlights several key points. First, the occurrence of Purtscher‐like retinopathy in young patients without trauma should prompt clinicians to investigate systemic causes, especially those involving vascular or inflammatory processes. Second, the role of supplement overuse as a possible precipitant emphasizes the need for awareness regarding the adverse systemic effects of nonprescribed substances, particularly anabolic steroids and excessive creatine intake, which can induce hypertension and renal impairment.

Currently, there is no established, evidence‐based treatment for Purtscher‐like retinopathy. Management primarily focuses on addressing the underlying systemic condition and providing supportive ocular care [1]. Some reports suggest that corticosteroids may have a role in reducing inflammation and edema, but evidence remains limited [2]. The prognosis varies; some patients experience spontaneous visual improvement, while others may have persistent deficits [2]. Here, systemic treatment targeted hypertension and renal failure without ocular interventions, yielding subjective improvement.

This case underscores the importance of interdisciplinary collaboration among ophthalmologists, internists, and nephrologists to ensure comprehensive management of patients presenting with Purtscher‐like retinopathy. Early recognition and control of systemic risk factors are essential to prevent further vascular damage and preserve visual function. Future research is warranted to better understand the pathogenic mechanisms and to develop targeted therapies for this rare but potentially sight‐threatening condition.

## Funding

The authors received no funding. It is the authors’ work, not funded by the government or academic institutes.

## Consent

Written informed consent was obtained from the patient for publication of this case report and any accompanying images.

## Conflicts of Interest

The authors declare no conflicts of interest.

## Data Availability

The authors confirm that the data supporting the findings of this study are available within the article.
